# Both a traditional and modified Daniel Fast improve the cardio-metabolic profile in men and women

**DOI:** 10.1186/1476-511X-12-114

**Published:** 2013-07-27

**Authors:** Rick J Alleman, Innocence C Harvey, Tyler M Farney, Richard J Bloomer

**Affiliations:** 1 Cardiorespiratory/Metabolic Laboratory, Department of Health and Sport Sciences, University of Memphis, 106 Roane Field House, Memphis, TN, USA

**Keywords:** Dietary modification, Nutrition, Reactive oxygen species, Lipids, Nitric oxide

## Abstract

**Background:**

The Daniel Fast involves dietary modification similar to a purified vegan diet. Although improvements in several health-specific biomarkers have been noted with this plan, the removal of animal products results in a significant reduction in both dietary protein and saturated fatty acid intake, which results in a loss of lean body mass and a reduction in HDL-cholesterol.

**Methods:**

We assigned 29 men and women to either a traditional or modified Daniel Fast for 21 days and measured anthropometric and biochemical markers of health pre and post intervention. The modified Daniel Fast was otherwise identical to the traditional plan but included one serving per day of lean meat and dairy (skim milk), providing approximately 30 grams per day of additional protein.

**Results:**

Compared to baseline, both plans resulted in similar and significant improvements in blood lipids, as well as a reduction in inflammation.

**Conclusions:**

Modification of dietary intake in accordance with either a traditional or modified Daniel Fast may improve risk factors for cardiovascular and metabolic disease.

## Background

For decades scientists have been investigating the effects of dietary intake on parameters of human health. One approach that has received a great deal of attention in recent years is the practice of fasting. Fasting is a period of abstinence from some or all food, drink, or both, usually for a pre-determined period of time (reviewed in [1]). Fasting plans that are frequently studied and presented in the scientific literature include caloric restriction (CR) [2], alternate day fasting (ADF) [3], and dietary restriction (DR) [4].

While both CR and ADF aim to decrease total caloric intake either daily or on an every- other-day cycle, DR typically involves *ad libitum* intake with restriction of selected nutrients or foods. The religiously-motivated Daniel Fast, a stringent vegan diet that has been investigated recently and noted to result in multiple health-related benefits [5,6], falls into the category of DR.

Specifically, the Daniel Fast is a plant-based nutritional plan inclusive of fruits, vegetables, whole grains, legumes, nuts, seeds, and healthy oils. No caffeine, alcohol, additives, or preservatives are allowed on the plan and participants usually partake in the plan for a period of 21 days—often as part of a religiously motivated fasting period. In our past work with the Daniel Fast, we have noted significant reductions in total cholesterol and LDL-cholesterol, but also a decrease in HDL-cholesterol [5]. Additional work involving a 21-day Daniel Fast has noted reductions in the total cholesterol/HDL-cholesterol ratio and LDL/HDL-cholesterol ratio, with similar effects on the other blood lipids [7].

Aside from blood lipids, we have noted a reduction in oxidative stress following the Daniel Fast. Specifically, we have observed a decrease in both malondialdehyde (MDA) and hydrogen peroxide (H_2_O_2_) in men and women [6]. We have also noted a significant and drastic reduction in systemic inflammation, as indicated by a decrease in C-reactive protein (CRP) [5,7]. Reducing systemic inflammation appears important, considering that increased inflammation and oxidative stress may be an underlying mechanism in the pathophysiology of aging and many age-related diseases [8-11]. Finally, the Daniel Fast results in a significant decrease in blood pressure and a small decrease in body weight (~ 6 pounds on average) and percent body fat (as measured via dual energy x-ray absorptiometry [DXA]).

The noted downside of the traditional plant-based Daniel Fast is a slight decrease in HDL-cholesterol [5], which may be problematic due to the importance of this cholesterol fragment in terms of providing atheroprotective benefits [12]. Moreover, with the decrease in body weight, we observe a slight decrease in lean body mass—amounting to approximately 50% of the overall weight loss. Identifying methods to minimize the decrease in both HDL-cholesterol and lean body mass are of interest and may make the Daniel Fast dietary approach of great interest to those seeking health benefits from their dietary plan.

One possible approach is the inclusion of small amounts of animal protein to the diet during a traditional Daniel Fast plan. Indeed, removing meat from the diet tends to lower HDL-cholesterol [13] and may result in a loss of lean body mass [14,15]. While some data indicate that meat consumption is directly related to an increased risk for disease [16], the quality of the meat (i.e., lean meat versus high-fat meat) needs to be considered when determining the potential impact on blood lipids and disease [17-19]. For example, diets that include moderate consumption of lean beef and chicken [20,21], as well as lean fish [22], are associated with modest increases in HDL-cholesterol and an overall improvement in the lipid profile. Related to factors aside from blood lipids, it appears that the protein from meat is not what causes an increase in oxidative stress and inflammation [23]. Rather, the type of fat appears to be more important when considering oxidative stress and inflammation, with increases in these variables noted following consumption of high amounts of saturated fatty acids (SFA) [24]. Therefore, consuming animal proteins that are relatively low in SFA may prove beneficial health wise.

Using a case study approach, we have noted that adding small quantities of low-fat meat (3–4 ounces of poultry or fish per day) and dairy (8 ounces of skim milk per day) allows for a similar reduction in total and LDL-cholesterol as compared to the traditional Daniel Fast, with little change in HDL-cholesterol. Moreover, the total cholesterol/HDL-cholesterol ratio was better with the modified plan compared to the traditional plan. Lean body mass decrements are also minimized with the addition of animal protein to the diet (unpublished observations, Bloomer). Using these initial data as a guide, the present study sought to determine the impact of a traditional and modified Daniel Fast on the blood lipid profile and other measures of cardio-metabolic health in a sample of otherwise healthy men and women.

## Results

Twenty-nine subjects completed all aspects of this study, with 13 in the modified Daniel Fast diet and 16 in the traditional Daniel Fast diet. Compliance was similar between groups (traditional: 96.0 ± 0.94% vs. modified: 91.4 ± 3.1%; p = 0.13). On a scale from 0–10, subjects rated their mood (mental outlook), vitality, and satiety during the diet. There were no condition x time interactions for the following variables (p > 0.05); “mental outlook” (traditional: 8.8 ± 0.3 vs. modified: 8.8 ± 0.3), “physical health and vitality” (traditional: 8.3 ± 0.5 vs. modified: 8.4 ± 0.5), and “feeling of satiety” (traditional: 8.8 ± 0.3 vs. modified: 8.3 ± 0.6). In both groups, “mental outlook” (p = 0.03) and “physical health and vitality” (p = 0.006) increased from pre to post fast (“mental outlook”: 8.1 ± 0.5 to 8.8 ± 0.3 and 7.9 ± 0.3 to 8.8 ± 0.3 in the traditional and modified groups, respectively; “physical health and vitality”: 7.4 ± 0.4 to 8.3 ± 0.5 and 6.6 ± 0.5 to 8.4 ± 0.5 in the traditional and modified groups, respectively).

### Anthropometric and blood pressure data

No condition x time interactions were noted for body weight (p = 0.98), total body fat percentage (p = 0.96), or trunk body fat percentage (p = 0.94). There was no significant difference in body weight from pre to post fast (p = 0.68) in the traditional group (pre: 81.7 ± 4.8 kg vs. post: 79.3 ± 4.9 kg) or in the modified group (pre 74.5 ± 6.1 kg vs. post: 72.4 ± 5.7 kg). Total body fat percentage was not significantly different (p = 0.92) from pre to post fast in the traditional group (pre: 35.7% ± 2.4 vs. post: 35.5 ± 2.5%) or in the modified group (pre: 33.8 ± 3.5% vs. post: 33.4 ± 3.6%). Similarly, trunk body fat percentage was not significantly different (p = 0.94) from pre to post fast in the traditional group (pre: 36.5 ± 2.5% vs. post: 36.2 ± 2.6%) or in the modified group (pre: 32.8 ± 3.7% vs. post: 32.0 ± 3.7%). Neither group experienced significant changes in fat mass (p = 0.80) or fat-free mass (p = 0.70) from pre to post fast. Resting heart rate or blood pressure was not significantly different from pre to post fast (p > 0.05). No other descriptive characteristic was different between diet plans or across time (p > 0.05; Table 1).

**Table 1:** Characteristics of men and women before and after a 21-day traditional or modified Daniel Fast

**Variable**	**Traditional**		**Modified**	
Time	Pre	Post	Pre	Post
Age (yrs)	42.4 ± 4.1	42.4 ± 4.1	41.7 ± 4.0	41.7 ± 4.0
Height (cm)	165.8 ± 2.0	165.9 ± 2.0	166.8 ± 2.2	166.8 ± 2.2
Weight (kg)	81.7 ± 4.8	79.3 ± 4.9	74.5 ± 6.1	72.4 ± 5.7
BMI (kg∙m^-2^)	29.7 ± 1.6	28.8 ± 1.6	26.6 ± 1.8	25.9 ± 1.7
Waist (cm)	90.2 ± 3.8	87.8 ± 4.0	82.6 ± 5.2	80.9 ± 4.9
Hip (cm)	109.8 ± 3.1	107.6 ± 3.1	106.0 ± 3.0	104.3 ± 2.9
Waist:Hip	0.82 ± 0.03	0.82 ± 0.03	0.77 ± 0.03	0.77 ± 0.03
DXA Total (%)	35.7 ± 2.4	35.5 ± 2.5	33.8 ± 3.5	33.4 ± 3.6
Fat-Free Mass (kg)	52.1 ± 3.3	50.6 ± 3.3	49.3 ± 4.1	48.2 ± 3.9
Fat Mass (kg)	29.6 ± 2.9	28.6 ± 2.9	26.4 ± 4.0	25.4 ± 3.9
DXA Trunk (%)	36.5 ± 2.5	36.2 ± 2.6	32.8 ± 3.7	32.0 ± 3.7
Heart Rate (bpm)	69.0 ± 2.2	65.6 ± 2.1	69.3 ± 2.5	68.3 ± 2.6
Systolic Blood Pressure (mmHg)	115.1 ± 5.0	111.9 ± 5.2	105.6 ± 3.9	106.7 ± 4.2
Diastolic Blood Pressure (mmHg)	72.2 ± 3.3	71.9 ± 4.3	68.9 ± 2.8	64.1 ± 2.7
Aerobic Exercise (hrs∙wk^-1^)	3.9 ± 1.0	NA	2.3 ± 0.4	NA
Years Aerobic Exercise	9.1 ± 3.2	NA	7.6 ± 2.3	NA
Anaerobic Exercise (hrs∙wk^-1^)	0.9 ± 0.3	NA	0.9 ± 0.4	NA
Years Anaerobic Exercise	2.0 ± 0.8	NA	4.3 ± 1.9	NA

Values are presented as mean ± SEM.

No statistically significant differences noted (p > 0.05).

### Biochemical data

The following differences were noted for complete blood count data: A condition effect was noted for white blood cells, with values higher for the traditional compared to the modified group (p = 0.02; 5.4×10^3^∙μU^-1^ vs. 4.6×10^3^∙μU^-1^). No other interaction, condition, or time effects of statistical significance were noted within the complete blood count (p > 0.05).

The following differences were noted for comprehensive metabolic panel data: A condition effect was noted for glucose, with values higher for the traditional compared to the modified group (p = 0.04; 100 mg∙dL^-1^ vs. 87 mg∙dL^-1^). A condition effect was also noted for protein, with values higher for the modified compared to the traditional group (p = 0.02; 6.9 g∙dL^-1^ vs. 6.6 g∙dL^-1^). A time effect was noted for potassium (p = 0.02; 4.5 mmol∙L^-1^ vs. 4.2 mmol∙L^-1^) and SGOT (p = 0.02; 24.9 IU∙L^-1^ vs. 19.2 IU∙L^-1^), with values higher post fast compared to pre fast. A time effect was also noted for BUN/creatinine (p = 0.04; 12.3 vs. 14.7), with values lower post fast compared to pre fast. No other interaction, condition, or time effects of statistical significance were noted within the comprehensive metabolic panel (p > 0.05).

No interaction, condition, or time effects of statistical significance were noted for any of the blood lipids (p > 0.05), except for total cholesterol. Total cholesterol significantly decreased (p = 0.02) to a similar extent in the traditional group (16.7%) and modified group (14.2%) but no interactions or condition effects were noted (p > 0.05). Although not of statistical significance, there was a greater reduction in HDL-cholesterol in the traditional Daniel Fast group (13.3%) compared to the modified Daniel Fast group (7.6%). No interaction, condition, or time effects of statistical significance were noted for CRP or HOMA-IR (p > 0.05). However, a reduction of 40% and 41% was noted for CRP in the traditional and modified groups, respectively. A reduction of 31% and 13% was noted for HOMA-IR in the traditional and modified groups, respectively. A condition effect was noted for insulin (p = 0.02), with values higher in the modified group compared to the traditional group. However, no interaction or time effects of statistical significance were noted for insulin (p > 0.05), despite a reduction of approximately 22% and 17% noted for the traditional and modified plans, respectively. A condition effect was noted for MDA (p = 0.03), with higher levels in the traditional group, but no significant interactions or time effects were noted (p > 0.05). A summary of our main outcome variables can be viewed in Tables 2 and 3.

**Table 2:** Oxidative stress and inflammatory data of men and women before and after a 21-day traditional or modified Daniel Fast

**Variable**	**Traditional**		**Modified**	
Time	Pre	Post	Pre	Post
MDA (μmol∙L^-1^)**	0.9 ± 0.1	0.7 ± 0.1	0.6 ± 0.0	0.6 ± 0.1
CRP (mg∙L^-1^)	1.4 ± 0.3	0.8 ± 0.2	1.5 ± 0.5	0.9 ± 0.3
Insulin (μU∙mL^-1^)**	13.6 ± 3.0	10.6 ± 2.1	7.0 ± 1.3	5.8 ± 0.9
HOMA-IR	4.2 ± 1.6	2.9 ± 0.9	1.5 ± 0.3	1.3 ± 0.2

Values are presented as mean ± SEM.

** Indicates significant difference between groups (MDA: p = 0.03; Insulin: p = 0.02).

No other statistically significant difference noted (p > 0.05).

**Table 3:** Lipid panel data of men and women before and after a 21-day traditional or modified Daniel Fast

**Variable**	**Traditional**		**Modified**	
Time	Pre	Post	Pre	Post
Cholesterol (mg·dL^-1^)	186.8 ± 13.5	155.6 ± 9.7*	180.7 ± 11.8	155.1 ± 11.9*
Triglycerides (mg·dL^-1^)	101.0 ± 10.9	78.7 ± 6.7	89.5 ± 8.9	79.3 ± 10.0
HDL-C (mg·dL^-1^)	56.3 ± 3.8	48.8 ± 3.4	58.6 ± 4.2	54.2 ± 3.7
VLDL-C (mg·dL^-1^)	20.2 ± 2.3	15.8 ± 1.3	18.1 ± 1.8	15.9 ± 2.0
LDL-C (mg·dL^-1^)	110.3 ± 12.1	91.1 ± 8.4	104.0 ± 10.9	85.1 ± 9.9
LDL-C:HDL-C	2.1 ± 0.2	2.0 ± 0.2	2.0 ± 0.3	1.7 ± 0.2
TC:HDL-C	3.5 ± 0.3	3.3 ± 0.2	3.3 ± 0.3	3.0 ± 0.3

Values are presented as mean ± SEM.

* Indicates significant difference from pre to post (p = 0.02).

No other statistically significant difference noted (p > 0.05).

### Dietary data

Dietary Data are presented in Table 4. Intake between the groups was significantly different for grams of protein (p = 0.009), percentage of protein (p = 0.001), selenium (p = 0.007), and calcium (p < 0.01). Compared to the traditional group, the modified group consumed significantly more protein (p = 0.04). During the diet, both groups consumed less kilocalories (p = 0.0002), grams of protein (p = 0.002), grams of carbohydrate (p < 0.01), sugar (p = 0.0006), grams of total fat (p < 0.0001), percentage of fat (p = 0.006), SFA (p < 0.0001), cholesterol (p < 0.0001), selenium (p < 0.01), and calcium (p < 0.0001), while consuming more grams of fiber (p = 0.0008), grams of soluble fiber (p = 0.01), percentage of carbohydrate (p = 0.018), vitamin A (p < 0.001), and vitamin C (p = 0.0005).

**Table 4:** Dietary data of men and women before and after a 21-day traditional or modified Daniel Fast

**Variable**	**Traditional**		**Modified**	
Time	Pre	Post	Pre	Post
Kilocalories	2070.3 ± 211.9	1141.1 ± 74.2*	1977.5 ± 186.7	1523.0 ± 203.8*
Protein (g)***	69.8 ± 5.3**	35.9 ± 2.7*	73.4 ± 9.7**	66.4 ± 6.8*
Protein (%)***	13.9 ± 0.5**	12.6 ± 0.7	15.1 ± 1.6**	18.5 ± 1.2
Carbohydrate (g)	256.6 ± 24.5	176.3 ± 13.0*	264.8 ± 31.7	206.2 ± 33.3*
Carbohydrate (%)	50.3 ± 2.0	62.3 ± 2.5	52.7 ± 2.3	53.9 ± 4.0
Fiber (g)	19.9 ± 2.0	26.8 ± 2.0*	18.5 ± 2.0	30.8 ± 4.4*
Fiber-soluble (g)	0.9 ± 0.4	2.4 ± 0.4*	1.3 ± 0.6	2.6 ± 0.8*
Sugar (g)	86.6 ± 9.7	53.9 ± 6.5*	110.4 ± 18.5	62.5 ± 7.4*
Fat (g)	84.2 ± 11.5	37.0 ± 3.9*	68.5 ± 8.0	43.8 ± 6.0*
Fat (%)	35.6 ± 1.7	28.5 ± 2.2*	31.5 ± 2.5	26.3 ± 2.1*
SFA (g)	25.9 ± 3.8	6.4 ± 0.7*	20.2 ± 2.6	7.4 ± 1.1*
Monounsaturated Fat (g)	21.3 ± 4.8	11.3 ± 1.8	15.3 ± 2.6	16.0 ± 2.4
PUFA (g)	9.7 ± 1.9	6.4 ± 0.8	9.2 ± 1.6	8.7 ± 1.2
Trans Fat (g)	3.1 ± 1.9	0.1 ± 0.1	1.7 ± 0.6	0.3 ± 0.2
Omega 3 (g)	0.6 ± 0.1	0.4 ± 0.1	0.5 ± 0.2	0.7 ± 0.1
Omega 6 (g)	7.8 ± 1.9	4.9 ± 0.7	5.9 ± 1.0	5.2 ± 0.7
Cholesterol (mg)	258.0 ± 38.0	12.3 ± 5.0*	219.0 ± 25.3	68.3 ± 11.5*
Vitamin C (mg)	44.9 ± 5.1	112.1 ± 21.7*	59.0 ± 12.0	109.7 ± 18.2*
Vitamin E (mg)	6.1 ± 1.2	5.4 ± 0.9	4.9 ± 0.9	7.2 ± 1.3
Vitamin A (RE)	208.8 ± 78.7	781.1 ± 155.8*	204.1 ± 49.5	584.3 ± 165.6*
Selenium (μg)	40.7 ± 5.2**	21.4 ± 2.8*	52.4 ± 8.5**	41.2 ± 5.4*
Calcium (mg)	583.6 ± 91.8**	251.4 ± 18.7*	700.2 ± 46.1**	464.4 ± 56.1*

Values are presented as mean ± SEM.

* Indicates significant difference from pre to post (p < 0.05).

** Indicates significant difference between groups (p < 0.05).

*** Indicates significant interaction (p < 0.05).

No other statistically significant difference noted (p > 0.05).

## Discussion

The results of the study indicate that there is no difference between a traditional Daniel Fast and a modified Daniel Fast, on selected markers of health. We hypothesized that the diets would yield similar decreases in MDA, CRP, insulin, and total and LDL-cholesterol. In addition, we hypothesized that the traditional Daniel Fast group would experience a greater reduction in HDL-cholesterol. Although slightly different, both diet plans resulted in noted improvements in several health-specific outcomes; however, only total cholesterol was noted to be of statistical significance. Other values (e.g., CRP, insulin, HOMA-IR, LDL-cholesterol, triglycerides) were decreased in a clinically meaningful manner but failed to reach statistical significance (p > 0.05). It is possible that our relatively small sample size did not provide adequate statistical power. Studies inclusive of a larger sample of subjects may be considered in future work with the Daniel Fast.

Prior work with the Daniel Fast, along with data from the present investigation, indicates that a Daniel Fast dietary regimen is beneficial for cardiovascular and metabolic health [5,6]. Our data indicate that these benefits are maintained when small quantities of animal products are added to a traditional Daniel Fast diet. In addition to the cardiovascular and metabolic benefits observed over a short period of time (21 days), the Daniel Fast has been shown to be well tolerated, with greater than 95% compliance [5,6]. However, we have noted that the restriction of animal products makes the diet somewhat challenging and less appealing to many individuals; this is especially true if individuals would like to adopt the diet plan long-term. While compliance was similar between the diets during the present investigation, further research is still needed to compare long-term compliance and health benefits. Our goal in the present study was to determine if the addition of animal products to a traditional Daniel Fast would produce similar results as seen in the past, when followed for the same duration of study (3 weeks). As indicated, this was the case.

Total cholesterol significantly decreased in both groups: 16.7% in the traditional group and 14.2% in the modified group. Both diet plans demonstrated a similar non-significant decrease in LDL-cholesterol, 17.4% and 18.2% in the traditional and modified groups, respectively. Moreover, both diets resulted in similar non-significant reductions in VLDL-cholesterol, triglyceride, LDL:HDL-cholesterol ratio, and total cholesterol:HDL-cholesterol ratio, which is similar to what has been observed in healthy men undergoing a vegetarian diet or a vegetarian plus lean meat diet [25]. We hypothesized that the traditional Daniel Fast group would experience a greater reduction in HDL-cholesterol, which is common when undergoing a vegan based diet [26]. Furthermore, low cholesterol diets that include moderate consumption of lean meat have shown modest increases in HDL-cholesterol in healthy [25] and hypercholesterolemic [20,22] subjects. It appears as though lean meat may not be responsible for the rise in cardiovascular related diseases; rather, additives, preservatives, processed nutrients, and the SFA associated with animal products and fast food may be the culprit.

In the present investigation, there was a non-significant decrease in HDL-cholesterol; 13.3% in the traditional group and 7.6% in the modified group. Earlier work with the traditional Daniel Fast demonstrated a significant reduction in HDL-cholesterol (14.5%) after 21 days of the diet [5]. As mentioned previously, adding small quantities of lean red or white meat to a cholesterol lowering diet helps maintain HDL-cholesterol in hypercholesterolemic men and women [20,22]. Likewise, significant increases in HDL-cholesterol, and the LDL:HDL-cholesterol ratio have been observed in healthy middle aged males undergoing a combination of a vegetarian diet and 150 g/day of lean meat for one month [25]. While the results indicate no difference of statistical significance between the diets, when adding a small amount of lean meat (3 ounces) and skim milk (8 ounces) to an otherwise strict vegan diet, less of a decrease in HDL-cholesterol is observed. Although not of statistical significance, the difference may have clinical relevance if the plan was carried out over a longer period of time and the changes were of greater magnitude—in particular in light of the fact that compliance to the dietary plan may be enhanced if subjects were allowed small amounts of meat and milk each day.

Some studies suggest that the omega-6/omega-3 fatty acid ratio is important when considering changes in HDL-cholesterol. In healthy young adult males, a 3% increase in HDL-cholesterol was observed after three weeks of a low SFA diet (9% of total energy) with increased intake of omega-3 polyunsaturated fatty acids (PUFA) (5 g/day) [27]. In another study, Rajaram et al. compared the effects of two diets rich in omega-3 PUFA and low in SFA (< 10% SFA) in mildly hyperlipidemic men and women, one from walnuts (42.5 g, 6 days/week) and the other from salmon (4 ounces raw, twice/week) [21]. After four weeks, the fish diet resulted in a significantly greater increase in HDL-cholesterol. However, a more favorable change in the LDL:HDL-cholesterol ratio was observed in the walnut diet. In the present investigation, the modified Daniel Fast group experienced a slightly smaller decrease in HDL-cholesterol compared to the traditional diet. Noteworthy, was the change in the LDL:HDL-cholesterol ratio. Although not significant, a 15.7% decrease was observed in the modified group, compared to a 5.8% decrease in the traditional diet. The omega-6/omega-3 fatty acid ratio in the traditional and modified groups was 12.3 and 7.4 respectively. With careful review of the changes in blood lipids, perhaps the modified group benefited from the combination of omega-3 PUFA from plants and meat. Nonetheless, a favorable change in the ratio is more important when considering the cholesterol carrying properties of the different lipoprotein particles, and may have clinical relevance.

In contrast to previous work with the Daniel Fast [6], in the present study MDA was not lowered in a statistically significant manner by either diet. Baseline MDA was higher in the traditional Daniel Fast group, whereas the modified group had relatively low baseline levels of MDA (0.92 ± 0.14 μmol∙L^-1^ vs. 0.62 ± 0.03 μmol∙L^-1^, respectively). While there were no significant differences between the groups from pre to post, a 16% decrease in MDA was observed in the traditional group whereas a 3.1% increase in MDA was observed in the modified group. Other studies have also shown that adding lean meat to the diet does not negatively impact biomarkers of oxidative stress. In a cohort study including healthy lacto-ovo-vegetarians and omnivores, MDA was not different across the groups [28]. When 215 g/day of lean red meat was used to replace carbohydrate rich foods in middle aged men and women with high blood pressure, plasma F_2_-isoprostanes did not change [23]. While it appears that small amounts of lean meat/protein does not negatively impact biomarkers of oxidative stress, diets that incorporate increasing amounts of fruit and vegetable intake have demonstrated favorable changes in oxidative stress biomarkers [23]. For example, urinary excretion of 8-isoprostane F_2α_ was significantly decreased in healthy women undergoing a high fruit and vegetable diet for eight weeks (9.2 servings of fruit and vegetables per day) [29]. Similarly, 8-isoprostane F_2α_ has been shown to significantly decrease after only 14 days of a dietary intervention that increased fruit and vegetable intake to 12 servings per day. Interestingly, the authors noted no difference in urine concentrations of MDA [30]. Isoprostanes and MDA are the more prominent biomarkers of lipid peroxidation. However, other relatively stable end products can be measured such as lipid hydroperoxides, α,β-unsaturated aldehydes, conjugated dienes, oxidized LDL, and 4-hydroxy-2-nonenal. In the present investigation, even though MDA did not change, it is possible that other biomarkers of oxidative stress were influenced. Using MDA as the exclusive biomarker of oxidative stress is a limitation of our study.

The ongoing regulation of the production and quenching of free radicals is an essential process in biological redox signaling. Dysfunction in the body’s ability to regulate reactive oxygen and nitrogen species (RONS) production has been implicated in the pathogenesis of many age related diseases; atherosclerosis, type II diabetes mellitus, and Alzheimer’s disease to name a few [31]. The inability to scavenge increased RONS production has also been implicated in the early onset of systemic inflammation [32]. Dietary intake can affect the inflammatory response in the body, and this response has been linked to oxidative stress, insulin resistance, obesity, and CVD [33]. C-reactive protein in particular has been shown to be a reliable marker of systemic inflammation during the development of CVD [34], and any effect on this biomarker by the two diet plans is noteworthy.

There were no observed differences in CRP between the two diet plans. In a population with already low CRP levels (< 1.5 mg/L), the two diet plans demonstrated a trend for reducing CRP, both producing greater than 40% reductions. Although not statistically significant, this change may be clinically meaningful, especially due to the fact that the American Heart Association has identified CRP as an independent predictor for CVD risk [35].

Both groups in the present investigation increased dietary antioxidant intake, specifically Vitamin C and Vitamin A. A crossover study in healthy middle aged men and women (~60 years old) undergoing a high-total antioxidant diet demonstrated significant decreases in CRP compared to a low-total antioxidant diet. In addition, no changes were observed in MDA following the dietary intervention [36]. The changes observed in the aforementioned study were similar to our findings in regards to the changes in CRP and MDA, indicating an improvement in systemic inflammation without changes in markers of oxidative stress. An investigation by Katcher et al. studied the effects of a hypocaloric diet that included whole grains, fruits, vegetables, and small quantities of lean meat and low-fat dairy products in obese individuals with metabolic syndrome. Sharing many of the same characteristics as a modified Daniel Fast, the results of the study indicated a significantly greater reduction in CRP when including whole grains compared to refined grains [37]. Others have shown SFA to be the most important variable contributing to CRP in healthy young adults [38]. This could explain a portion of the benefits observed in the present investigation, owing to the fact that both groups reduced dietary SFA to less than 8 grams/day during the intervention.

Recently, large cohort studies have delineated a relationship between red meat consumption, metabolic syndrome [39,40] and increased mortality [40]. Included in these cohort studies are processed meats, organ meats, and red meats. Indeed, the consumption of processed meats have been associated with coronary heart disease and diabetes, however, this relationship does not hold true for red meat alone (i.e., beef, hamburgers, lamb, pork, or game) [41]. Based on our investigation, as well as a review of the relevant literature, the effects of adding a moderate amount of lean meat (i.e., red meat, white meat, and/or fish), in addition to one serving per day of skim milk, to a strict vegan diet produces similar results in health biomarkers as a strict vegan diet. This is evidenced not only by the noted changes in CRP and cholesterol fragments, but also by the reduction in insulin and HOMA-IR in both diet groups.

## Conclusion

In conclusion, our results indicate that the addition of lean meat and skim milk to a traditional Daniel Fast appears as beneficial as the vegan-based Daniel Fast, if not yielding slightly more favorable results with regard to changes in HDL-cholesterol and certain lipid panel ratios. Both diets produced similar changes in all measured outcome variables. Future research is needed to assess the long-term health benefits of these plans, as well as the long-term compliance of individuals following these approaches within a free- living environment.

## Methods

Twenty-nine healthy men and women between the ages of 18 and 66 years were enrolled in this study and completed all requirements. Subjects were not current smokers, and did not have a history of cardiovascular disease (e.g., high blood pressure). Most subjects were physically active, engaged in regular exercise programs. Health history, medication and dietary supplement usage, and physical activity questionnaires were completed by all subjects. Prior to participation, each subject was informed of all procedures, potential risks, and benefits associated with the study through both verbal and written form in accordance with the procedures approved by the University Institutional Review Board for Human Subjects Research (Approval #702). All subjects provided written informed consent prior to being admitted as a subject.

### Study timeline

Week 0 Initial screening: Paperwork and diet instructions/logs; diet plan assignment.

Week 1 Complete 7 day diet record and prepare for diet plan.

Week 2–4 Day 1: Fasting blood sample, measurements; Start diet plan (for 3 weeks).

Week 4: Complete 7 day diet record.

End Week 4: Fasting blood sample, measurements, and questionnaire.

### Traditional and modified Daniel fast

Subjects were assigned to either a traditional Daniel Fast (vegan diet) or a modified Daniel Fast inclusive of one serving per day of meat (3 ounces of chicken, fish, beef, pork, or turkey) and skim milk (8 ounces). Following all baseline assessments and the initial week of dietary recording, subjects underwent their Daniel Fast assignment for 3 weeks. Subjects were provided an outline of the foods that are routinely allowed, as well as commonly consumed foods that are not allowed. They were also given websites that they could refer to for more specifics. For simplicity sake, subjects were informed that the diet plan essentially consists of fruits and vegetables, nuts, seeds, legumes, vegetables, oil, and whole grains. In addition, no additives, preservatives, flavoring, caffeine, or alcohol were allowed. Subjects were allowed decaffeinated coffee and tea, as well as water, to drink. This applied to subjects in both plans (traditional and modified). Those subjects assigned to the traditional Daniel Fast plan were not allowed any animal products, while subjects assigned to the modified Daniel Fast plan were *required* to consume one serving per day of lean meat (3 ounces) and one serving per day of skim milk (8 ounces). Subjects were exposed to food models to have a better understanding of portion sizes.

### Lab visits

During the initial visit to the lab, subjects completed the informed consent form, health, and physical activity questionnaires. They were also informed of their assigned condition (traditional or modified Daniel Fast plan), and received study instructions and a study schedule. During the other lab visits (end of weeks 1 and 4), all assessments were performed. Heart rate (via palpation) and blood pressure (via cuff and stethoscope) was recorded following a 10 minute period of quiet rest, in a seated position. A blood sample was then taken from subjects, as described below. Subjects’ height, weight, and circumference measures were recorded. Subjects also had a scan performed to determine their body composition (via DXA) using a Hologic QDR 4500 W scanner.

### Blood collection and biochemistry

Venous blood samples (approximately 20 mL) were taken from subjects via needle and Vacutainer®. Blood samples were collected following an overnight fast (10 hours) in a rested state on day one of the fast and on the day following completion of the fast. Following collection, blood collected in tubes with no additives was allowed to clot at room temperature for 30 minutes and then separated to serum by centrifugation at 1500 g for 15 minutes at 4°C. Blood collected in tubes containing ethylenediaminetetraacetic acid (EDTA) was used for analysis as indicated below, or immediately separated to plasma by centrifugation at 1500 g for 15 minutes at 4°C.

Fresh blood samples were analyzed for complete blood count using an automated cell counter (Coulter LH750). A comprehensive metabolic panel was determined using automated procedures (Roche/Hitachi Modular). A lipid panel was determined using enzymatic procedures (Roche/Hitachi Modular). As a measure of systemic inflammation, high-sensitivity CRP was determined using a high-sensitivity, particle-enhanced turbidimetric immunoassay (Roche Integra 800). Insulin was determined using an immuno-chemiluminescent assay procedure (Roche Modular E170). The homeostasis model assessment (HOMA-IR) was used as an index of insulin resistance [42] and calculated as: [fasting glucose (mg∙dL^-1^) × fasting insulin (μU∙mL^-1^)] / 405. Frozen blood samples were thawed once, mixed thoroughly, and analyzed for MDA. The procedures of Jentzsch et al. [43] were followed using reagents purchased from Northwest Life Science Specialties (Vancouver, WA), with quantification performed using a tetramethoxypropane standard curve.

### Dietary records and activity

During the initial lab visit a full explanation of dietary recording was provided to subjects, along with data collection forms. An overview of study procedures was also provided. Subjects were instructed to maintain their normal diet during week one of the study (prior to beginning the fast) and to record on data forms all food and drink consumed. During week 2 of the study, subjects began the Daniel Fast (traditional or modified) and were asked to record on data forms all food and drink consumed during week 4 of the study (the final week of the fast). Nutritional records from subjects before and during the fast period were analyzed (Food Processor SQL, version 9.9, ESHA Research, Salem, OR). Subjects were instructed to maintain their normal physical activity habits during the entire study period. Subjects were given specific instructions regarding abstinence of alcohol consumption, in addition to the avoidance of strenuous exercise during the 48 hours immediately before the assessment days.

### Compliance, mood, vitality, and satiety

As an assessment of compliance, mood (mental outlook), vitality, and satiety, subjects were asked to complete a questionnaire related to their experience on the Daniel Fast. Subjects were asked to rate their compliance to the dietary guidelines on a percentage basis, from 0% compliant to 100% compliant. For mood, vitality, and satiety, subjects rated each item using a scale of 1–10 (1 = as low as possible; 10 = as high as possible). These data were based solely on subjects’ self-report.

### Statistical analysis

Data were analyzed using a 2 (dietary plan) × 2 (time) analysis of variance (ANOVA). Tukey post hoc tests were used as needed. Compliance, mood, vitality, satiety, and dietary data were analyzed using a one-way ANOVA. All analyses were performed using JMP statistical software (version 4.0.3, SAS Institute, Cary, NC). Statistical significance was set at P ≤ 0.05. The data are presented as mean ± SEM.

## Competing interests

The authors declare no competing interests relative to this work.

## Authors’ contributions

RJA, ICH, and TMF were responsible for subject recruitment, screening and retention, data collection and entry, and blood collection and processing. ICH performed the DXA scans. RJB was responsible for the study design, oversight and analysis of biochemical variables, and statistical analyses. RJA and RJB wrote the manuscript. All authors reviewed and approved of the final manuscript.
